# Supplementary material for: Analysis of PPARγ Signaling Activity in Psoriasis
Source: Int J Mol Sci. 2021 Aug 10;22(16):8603. doi: 10.3390/ijms22168603 (PMC8395241; doi:10.3390/ijms22168603)
Supplement: Supplementary file 1 [file ijms-22-08603-s001.zip › Supplemental materials_Analysis of PPARg signaling activity in psoriasis/Pathway models/Models images and html files/Interleukin-17 and interleukin-22 signaling in psoriasis/9721.html]

RAF1


# Protein RAF1

|  |  |
| --- | --- |
| URN | urn:agi-llid:5894 |
| Total Entities | 0 |
| Connectivity | 4428 |
| Name | RAF1 |
| Description | v-raf-1 murine leukemia viral oncogene homolog 1 |
| Notes | This gene is the cellular homolog of viral raf gene (v-raf). The encoded protein is a MAP kinase kinase kinase (MAP3K), which functions downstream of the Ras family of membrane associated GTPases to which it binds directly. Once activated, the cellular RAF1 protein can phosphorylate to activate the dual specificity protein kinases MEK1 and MEK2, which in turn phosphorylate to activate the serine/threonine specific protein kinases, ERK1 and ERK2. Activated ERKs are pleiotropic effectors of cell physiology and play an important role in the control of gene expression involved in the cell division cycle, apoptosis, cell differentiation and cell migration. Mutations in this gene are associated with Noonan syndrome 5 and LEOPARD syndrome 2. [provided by RefSeq, Jul 2008] |
| Primary Cell Localization | Cytoplasm |
| Class | Protein kinase |

---

|  |  |
| --- | --- |
| Pathway | Alzheimer's Disease |
|  | Osteoarthritis |
|  | Skin Aging |
|  | Oncogene Induced Cellular Senescence |
|  | Melatonin Effects on Circadian Cycle |
|  | mTOR Signaling |
|  | mTOR Signaling Hyperfunction |
|  | High Fat Diet Activates cAMP Related Exocytosis in Synapse and Endocrine Cell |
|  | mTOR Signaling Activation by Taste Sensor Receptors (Rodent Model) |
|  | Insulin Influence on Protein Synthesis |
|  | Insulin Influence on Glycogenesis |
|  | Dopamine Receptors Signaling |
|  | Serotonin Receptors Signaling |
|  | Thyroid Hormones Common non-Genomic Effects in Hyperthyroidism |
|  | CRH Secretion Regulation |
|  | Insulin Signaling |
|  | Prolactin Production |
|  | Thrombopoietin Receptors Signaling in Platelet Maturation |
|  | Aldosterone Renal Effects |
|  | Dectin-1 (CLEC7A) Signaling |
|  | DC-SIGN (CD209) Signaling |
|  | T-Cell Dependent B-Cell Activation |
|  | T-Cell Independent B-Cell Activation |
|  | Natural Killer Cell Activation through C-Type Lectin-like Receptors |
|  | Natural Killer Cell Activation through ITAM-Containing Receptors |
|  | Natural Killer Cell Activation through ITSM-Containing Receptors |
|  | Natural Killer Cell Activation through Integrins and non-ITAM-Containing Receptors |
|  | T-Cell Maturation (Hypothesis) |
|  | Natural Killer Cell Activation |
|  | CD8+ T-Cell Activation |
|  | Th1-Cell Differentiation |
|  | Treg-Cell Differentiation |
|  | Th2-Cell Differentiation |
|  | T-Cell Receptor Signaling |
|  | CXC Chemokine Receptor Signaling |
|  | CC Chemokine Receptor Signaling |
|  | Plasmin Effects in Inflammation |
|  | Platelet Activation via Adhesion Molecules |
|  | Proplatelet Maturation |
|  | Platelet Activation via GPCR Signaling |
|  | Toll-like Receptor Independent Sterile Inflammation |
|  | Lipoxin A4/FPR2-Related Neutrophil Depression |
|  | MC1R in anti-Inflammatory Signaling |
|  | Eicosanoids in Myeloid Cell Activation |
|  | Leukotriene Effect on Airway Smooth Muscle Response |
|  | Neutrophil Activation via LTB4/Polyisoprenyl Phosphate Signaling |
|  | Basophil Activation |
|  | Eosinophil Survival by Cytokine Signaling |
|  | Eosinophil Activation |
|  | Mast-Cell Activation via IgE Signaling |
|  | Mast-Cell Activation without Degranulation through CYSLTR1/CYSLTR2 Signaling |
|  | Mast-Cells De Novo Synthesized Mediators via IgE Independent Signaling |
|  | CD16/CD14 Proinflammatory Monocyte Activation |
|  | Macrophage M2-Related Phagocytosis |
|  | anti-Inflammatory Function of Macrophage M2 Lineage |
|  | Neutrophil Recruitment and Priming |
|  | Neutrophil Activation via FCGR3B |
|  | Neutrophil Degranulation via FPR1 Signaling |
|  | Neutrophil Chemotaxis |
|  | Neutrophil Activation via Adherence on Endothelial Cells |
|  | Negative Acute Phase Proteins Synthesis |
|  | Positive Acute Phase Proteins Synthesis |
|  | Vascular Smooth Muscle Cell/Pericyte Migration and Proliferation |
|  | Vascular Endothelial Cell Activation by Blood Coagulation Factors |
|  | Vascular Endothelial Cell Activation by Growth Factors |
|  | Vascular Endothelial Cell Permeability Activation |
|  | Nociception Expression Targets Signaling |
|  | Vascular Motility |
|  | NTSR1 Expression Targets |
|  | EDNRA/B -> Vascular Motility |
|  | ADRA1 -> Vasoconstriction |
|  | ADRA1A -> IL6 Production |
|  | ADRA1 -> Prostaglandin Generation |
|  | BDKRB1/2 -> Prostaglandin Generation |
|  | DRD2 Expression Targets |
|  | GALR1/2/3 -> POMC/NPY Production |
|  | HRH2/4 -> IL6/10 Production |
|  | CHRM1/2/3 -> Vascular Motility |
|  | OPRK -> Pain Perception |
|  | PTGIR -> IL6 Production |
|  | PTGER1/4 -> Vascular Motility |
|  | PTGER2/3 -> Inflammation-Related Expression Targets |
|  | IL6 Expression Targets -> Nociception |
|  | Luteinizing Hormone in Oocyte Meiotic Resumption |
|  | Melatonin in Cell Survival and Antioxidant Response |
|  | Vitamine D Deficite and Dentin Formation |
|  | non-Genomic Rapid Actions of Vitamin D in Vitamin D Biology |
|  | G0/G1 Phase Transition |
|  | Scavenger Receptor OLR1 in Inflammation Related Endothelial Dysfunction |
|  | RAGE/AGER and S100 Proteins in Cardiovascular Injury |
|  | Th1-Cells Activation and Proliferation in Atherosclerosis |
|  | Proteins Involved in Hypertrophic Cardiomyopathy |
|  | Cardiomyocyte Hypertrophy |
|  | Hypertrophic Cardiomyopathy |
|  | Aldosterone Signaling in Arterial Hypertension |
|  | TRPM4/6/7/8 Signaling in Arterial Hypertension (Hypothesis) |
|  | Proteins Involved in Pulmonary Hypertension |
|  | Smooth Muscle Cell Dysfunction in Pulmonary Hypertension |
|  | Myocardial Remodeling |
|  | Contraction Due Vasospasm |
|  | Sebocyte Proliferation in Acne Vulgaris |
|  | Inflammatory Reaction in Acne Vulgaris |
|  | Hair Follicle Keratinocyte Apoptosis |
|  | Atopic Dermatitis Onset |
|  | Mast-Cells Activation in Atopic Dermatitis |
|  | Cell Cycle Dysregulation in Melanoma |
|  | Melanoma |
|  | Apoptosis Block in Melanoma Cell |
|  | MITF as a Regulator of Melanoma Cell Development |
|  | Proteins Involved in Melanoma |
|  | IL22 Induces Keratinocyte Proliferation in Psoriasis |
|  | Th0-Cell Aberrant Activation in Systemic Lupus Erythematosus |
|  | Th1-Cell Function in Systemic Lupus Erythematosus |
|  | B-Cell Activation in Systemic Lupus Erythematosus |
|  | Th2-Cell Function in Systemic Lupus Erythematosus |
|  | Acute Cytotoxic CD8+T-Cell Response against Melanocytes |
|  | Vitiligo |
|  | T-Cell Cytotoxic Response against Melanocytes in Vitiligo |
|  | Apoptosis of Melanocyte in Vitiligo |
|  | Humoral Immunity in Vitiligo |
|  | CD8+ T-Cell Response to Self-Determinants in Diabetes |
|  | Treg-Cell Function in Diabetes Mellitus Type 1 |
|  | Natural Killer T-Cell Roles in Diabetes Mellitus Type 1 |
|  | non-Suppressive Treg-Cell in Diabetes Mellitus Type 1 |
|  | TLR2 Signaling in Treg-Cell in Type 1 Diabetes (Animal Model) |
|  | Natural Killer Cell in Diabetes Mellitus Type 1 |
|  | Peripheral Tolerance to Autoantigens Recession in Diabetes Mellitus Type 1 |
|  | Treg-Cell Activation in Diabetes Mellitus |
|  | Donohue Syndrome Progression (Hypothesis) |
|  | Advanced Glycation End Products Pathway in Diabetic Microangiopathy |
|  | Protein Kinase C in Diabetic Microangiopathy |
|  | Peripheral Tissue Microangiopathy |
|  | Microangiopathy in Diabetic Neuropathy |
|  | Proliferative Diabetic Retinopathy |
|  | AGE Related Insulin Resistance in Skeletal Muscles |
|  | Adiponectin Synthesis Declines in Insulin Resistance |
|  | Hepatic Stellate Cells in non-Alcoholic Fatty Liver Disease |
|  | FOXO1 and SREBP-1C Role in beta-Cell Suppression (Rodent Model) |
|  | Hyperparathyroidism, Neonatal Severe |
|  | RET1 Mutation in Hyperparathyroidism |
|  | Hypothyrodism Associated Hyperprolactinemia |
|  | Ethanol Induced Efects in Hyperprolactinemia |
|  | Bone Remodeling in Hyperthyroidism |
|  | Cardiovascular Effects in Hyperthyroidism |
|  | Thyroid Dysfunction |
|  | GNRH Signaling Impairment in Hypogonadotrophic Hypogonadism |
|  | Thyroid Stimulating Hormone (TSH) Resistance in Congenital Hypothyroidism |
|  | Overt Hypothyroidism, Primary |
|  | Thyroid Stimulating Hormone (TSH) Secretion in Overt Hypothyroidism |
|  | Prolactinoma Overiew |
|  | CD4+T-Cell Response in Celiac Disease |
|  | CD8+ T-Cell Response in Celiac Disease |
|  | Celiac Disease |
|  | Metastatic Colorectal Cancer |
|  | OipA Signaling in Helicobacter Pylori Infection |
|  | CagA Phosphorylation Dependent Signaling |
|  | EDN, NRG, NRTN, and GDNF/RET Signaling in Hirschsprung Disease |
|  | Th2-Cell Function in Ulcerative Colitis |
|  | T-Cell Cytotoxic Response in Ulcerative Colitis |
|  | FLT3/KIT/MLL Signaling in Leukemia |
|  | Acute Myeloid Leukemia |
|  | Myeloid Cell Differentiation Block in Leukemia |
|  | B-Cell Acute Lymphoblastic Leukemia |
|  | B-Cell Chronic Lymphocytic Leukemia |
|  | Chronic Myeloid Leukemia |
|  | Follicular Lymphoma |
|  | Hodgkin Lymphoma |
|  | Mucosa-Associated Lymphoid Tissue (MALT) Lymphoma |
|  | IL6/IGF1/VEGFA Signaling in Multiple Myeloma |
|  | Multiple Myeloma |
|  | T-Cell Acute Lymphoblastic Leukemia |
|  | TRPM7/8 in Pancreatic Adenocarcinoma |
|  | Il17 Signaling Related Neutrophilia in Asthma |
|  | Eosinophil Activation and Degranulation in Asthma |
|  | IL17F Signaling in Bronchial Epithelial Cell in Asthma |
|  | Airway Smooth Muscle Cell High Rate Proliferation |
|  | IgE Induces Airway Smooth Muscle Cell Proliferation |
|  | Goblet-Cell Related Mucus Secretion in Asthma |
|  | Neutrophil Degranulation via FPR1/IL8 |
|  | Basophil Activation in Asthma |
|  | Mast-Cell Activation in Asthma |
|  | Antigen-Presenting Cell Role in Asthma |
|  | Th2-Cell Response in Asthma |
|  | Mucin Production in Goblet Airway Epithelial Cells |
|  | Osteoclast Activation in Gout |
|  | TNF and IL1B Induce Metalloproteinase Synthesis in Osteoarthritis |
|  | Steroidogenesis Impairement in Polycystic Ovary Syndrome |
|  | Proteins Involved in Polycystic Ovary Syndrome |
|  | Polycystic Ovary Syndrome |
|  | PTPN22 Role in Rheumatoid Arthritis |
|  | Synovial Fibroblast Proliferation in Rheumatoid Arthritis |
|  | Osteoclast Activation in Rheumatoid Arthritis |
|  | B-Cells Function in Systemic Scleroderma |
|  | Th2-Cells Function in Systemic Scleroderma |
|  | Skin Fibrosis |
|  | Dentin Formation Disruption |
|  | CD8+ T-Cell Action Impairment in HIV Type 1 Infection |
|  | CD4+ T-Cell Function Decline in HIV |
|  | CD4+ T-Cell Death in HIV |
|  | IL1 Receptor Antagonist (IL1RN) Deficiency (DIRA) |
|  | Cowden Syndrome |
|  | Gluten Impact on Neuronal System (Hypothesis) |
|  | Osteoblast Function Decline in Osteoporosis |
|  | Osteoclast Activation in Postmenopause |
|  | BDNF Upregulation Triggered by Seizures |
|  | Apoptosis and Immediate Early Gene Activation in Epileptiform Disorders |
|  | Synaptic Exocytosis in Dementia |
|  | Glioblastoma, Primary |
|  | Proteins Involved in Glioblastoma |
|  | Glioblastoma, Secondary |
|  | Astrocytoma |
|  | Glioma Invasion Signaling |
|  | Proteins Involved in Glioma |
|  | Growth Factor Signaling in Neuroblastoma |
|  | Neuroblastoma |
|  | Proteins Involved in Breast Cancer Related to ESR1 Signaling Pathway |
|  | Proteins Involved in Breast Cancer Related to ERBB2/VEGFR/Akt Signaling Pathway |
|  | ERBB/VEGFR/Akt Signaling in Breast Cancer |
|  | IGF1R/AKT Signaling in Breast Cancer |
|  | ESR1/ERBB Positive Luminal Breast Cancer |
|  | Proteins Involved in Breast Cancer Related to IGF1R/Akt Signaling Pathway |
|  | Breast Cancer |
|  | ESR1 Signaling in Breast Cancer |
|  | Proteins Involved in Endometriosis |
|  | Angiogenesis in Endometriosis |
|  | Coagulation Factors and PAR1/2 Receptors (F2R/F2RL1) in Endometriosis |
|  | Leptin Role in Endometriosis |
|  | Proteins Involved in High-Grade Serous Ovarian Carcinoma |
|  | Proteins Involved in Endometrioid Ovarian Carcinoma |
|  | Proteins Involved in Mucinous Ovarian Carcinoma |
|  | EGFR and PTAFR Signaling in Ovarian Cancer |
|  | Proteins Involved in Low-Grade Serous Ovarian Carcinoma |
|  | Proteins Involved in Clear Cell Ovarian Carcinoma |
|  | HMGB1/RAGE Signaling in Middle Ear Cholesteatoma |
|  | Subepithelial Fibroblasts in Middle Ear Cholesteatoma |
|  | Cholesteatoma |
|  | HPV E5 Regulates EDNRA and EGFR Signaling Pathway in Keratinocytes |
|  | Prostate Cancer |
|  | TRPM8 Effects in Prostate Cancer (Hypothesis) |
|  | Proteins Involved in Prostate Cancer |
|  | Steroids Induced Cataract |
|  | VEGFA in Neovascular Glaucoma |
|  | PGF2-alpha Effect on Treatment |
|  | Cytokines Trigger Otitis Media |
|  | VEGFA Low Expression Level in Chronic Bronchitis |
|  | VEGFA High Expression Level in Pulmonary Emphysema |
|  | Alveolar Epithelial Cell Dysfunction in COPD |
|  | Mucin Hyperproduction in Goblet and Mucous Cells |
|  | Polycystins Mutation Effects in Cystic Kidney Disease |
|  | Proteins Involved in Polycystic Kidney Disease |
|  | Plasmin Role in Glomerulonephritis |
|  | Endothelial Cell Dysfunction in Glomerulonephritis |
|  | Sertoli Cells Dysfunction Causes Male Infertility |
|  | CD38/CD3 -> JUN/FOS/NF-kB Signaling in T-cell Proliferation |
|  | CD38/CD19 -> JUN/FOS/NF-kB Signaling in B-cell Proliferation |
|  | CD38 -> cADPR/Calcium Signaling |
|  | CD157 -> cADPR/Calcium Signaling |
|  | S100P Expression Target |
|  | HMGB1 Expression Targets |
|  | TTR Expression Targets |
|  | S100A Expression Targets |
|  | S100B Expression Targets |
|  | ANGPT1 -> CREB/CREBBP Expression Targets |
|  | ANGPT2 -> AP-1/CREBBP/MYC Expression Targets |
|  | AREG -> CREB/CREBBP Expression Targets |
|  | AREG -> NCOR2 Expression Targets |
|  | AREG -> SMAD1 Expression Target |
|  | AREG -> HIF1A Expression Targets |
|  | AREG -> AP-1 Expression Targets |
|  | Noreadrenaline/Gq Expression Targets |
|  | Serotonin/Gq Expression Targets |
|  | Epinephrine/Gq Expression Targets |
|  | Dopamine/Gi Expression Targets |
|  | BTC -> AP-1/ATF/CREB Expression Targets |
|  | BTC -> EP300/ETS/ETV/SP1 Expression Targets |
|  | SELE -> ELK-SRF Signaling |
|  | CD19 Expression Targets |
|  | CD81 -> AP-1 Expression Targets |
|  | CD247 Expression Targets |
|  | FCGR3A Expression Targets |
|  | CD19/CR2 Expression Targets |
|  | CD72 -> CREB/CREBBP Expression Targets |
|  | CD8 -> ATF/CREB/CREBBP Expression Targets |
|  | CD80 -> NF-kB Expression Targets |
|  | CD86 -> NF-kB Expression Targets |
|  | EGF -> HIF1A Expression Targets |
|  | EGF -> AP-1/ATF Expression Targets |
|  | EGF -> CREB/CREBBP/ELK/SRF/MYC Expression Targets |
|  | EGF -> TP53 Expression Targets |
|  | EGF -> NCOR2 Expression Target |
|  | EGFR -> NCOR2 Signaling |
|  | EGFR/ERBB2 -> TP53 Signaling |
|  | ERBB2/3 -> EP300/ETS/ETV/SP1 Signaling |
|  | EGFR -> SMAD1 Signaling |
|  | EGFR/ERBB2 -> HIF1A Signaling |
|  | EGFR -> AP-1/ATF2 Signaling |
|  | EGFR -> AP-1/CREB/ELK/SRF/MYC Signaling |
|  | EREG -> CREB Expression Target |
|  | EREG -> EP300/SP1 Expression Targets |
|  | EREG -> HIF1A Expression Target |
|  | EREG -> AP-1/ATF Expression Targets |
|  | Erythropoietin -> AP-1/MYC/CREB Expression Targets |
|  | Erythropoietin -> ELK/SRF Expression Targets |
|  | PECAM1 Expression Targets |
|  | NCAM1 Expression Targets |
|  | F2 -> AP-1/CREB/ELK/SRF/SP1 Expression Targets |
|  | PLG -> AP-1/CREB/ELK/SRF/SP1 Expression Targets |
|  | FGF9 -> AP-1/CREB/MYC Expression Targets |
|  | FGF10 -> AP-1/CREB/CREBBP/MYC Expression Targets |
|  | FGF1 -> AP-1/CREB/ELK/SRF/MYC Expression Targets |
|  | FGF1 -> NCOR2 Expression Target |
|  | FGF7 -> RUNX Expression Targets |
|  | FGF8 -> AP-1/CREB/MYC Expression Targets |
|  | FGF1 -> RUNX Expression Targets |
|  | FGF18 -> AP-1/CREB Expression Targets |
|  | FGF23 -> NCOR2 Expression Targets |
|  | FGF8 -> RUNX Expression Targets |
|  | FGF9 -> RUNX Expression Targets |
|  | FGF2 -> NCOR2 Expression Targets |
|  | FGF4 -> AP-1/MYC Expression Targets |
|  | FGF7 -> AP-1/CREB/CREBBP/MYC Expression Targets |
|  | FGF2 -> RUNX Expression Targets |
|  | FGF2 -> AP-1/CREB/CREBBP/ELK/SRF/MYC Expression Targets |
|  | FGFR -> RUNX2 Signaling |
|  | FGFR -> AP-1/CREB/CREBBP/ELK/SRF/MYC Signaling |
|  | GH1/GHR -> NF-kB/ELK/SRF/MYC Expression Targets |
|  | CSH1/GHR Expression Targets |
|  | HBEGF -> AP-1/ATF Expression Targets |
|  | HBEGF -> TP53 Expression Targets |
|  | HBEGF -> EP300/ETS/ETV/SP1 Expression Targets |
|  | HBEGF -> HIF1A Expression Targets |
|  | HBEGF -> CREB/MYC Expression Targets |
|  | HGF -> AP-1/CREB/ELK/SRF/MYC Expression Targets |
|  | HGFR -> AP-1/CREB/MYC Signaling |
|  | TAM Receptor Signaling Activation in Cancer |
|  | G0/G1 Cell Cycle Phase Transition Activation in Cancer |
|  | RAS/RAF/MAPK Signaling Activation in Cancer |
|  | RAS/RAF/MAPK Signaling Activation by Blocking of Tumor Suppressors |
|  | HIF1A in Vasculogenic Mimicry of Cancer |
|  | VEGFA Dependent Angiogenesis in Cancer |
|  | ANGPT/TEK Stimulates Endothelial Cell Migration in Cancer |
|  | VEGF Independent Angiogenesis in Cancer |
|  | Integrins in Cancer Cell Motility, Invasion and Survival |
|  | MTOR/TORC in Cancer Cell Motility and Invasion |
|  | CDH2 Activation Promotes Cancer Cell Migration and Survival |
|  | Lactate as a Signaling Molecule in Cancer Cells |
|  | Treg Cells Promote Immunosuppression in Cancer Immune Escape |
|  | EZH2 Methyltransferase Role in Cancer |
|  | IGF2 -> HIF1A/MYC Expression Targets |
|  | IGF1 -> ELK/SRF/HIF1A/MYC/SREBF Expression Targets |
|  | Insulin -> ELK/SRF/HIF1A/MYC/SREBF Expression Targets |
|  | AGER -> CREB/SP1 Signaling |
|  | NCAM1 -> CREB/ELK/SRF/MYC Signaling |
|  | CD19 -> JUN/ELK Signaling |
|  | PECAM -> SP1 Signaling |
|  | FcIgER -> ELK-SRF Signaling |
|  | ICAM1 -> AP-1/CREB/ELK/SRF Signaling |
|  | IGF1R -> ELK/SRF/HIF1A/MYC/SREBF Signaling |
|  | InsulinR -> ELK/SRF/SREBF Signaling |
|  | FibronectinR -> AP-1/ELK/SRF/SREBF Signaling |
|  | CTGF -> AP-1/CREB/MYC Expression Targets |
|  | CTGF -> NCOR2 Expression Target |
|  | ICAM1 Expression Targets |
|  | IL6R -> CEBP/ELK/SRF Signaling |
|  | IL2R -> ELK/SRF/MYC Signaling |
|  | IL4R -> ELK/SRF/HMGY Signaling |
|  | IL16 -> AP-1 Expression Targets |
|  | IL6 Expression Targets |
|  | IL4 Expression Targets |
|  | IL2 Expression Targets |
|  | UrokinaseR -> ELK/SRF Signaling |
|  | PTPRC -> BCL6 Expression Targets |
|  | Leptin -> ELK/SRF Expression Targets |
|  | Prostaglandin F Expression Targets |
|  | LPA Expression Targets |
|  | Morphine Expression Targets |
|  | PGE1 Expression Targets |
|  | MTNR1 Signaling |
|  | NRG1 -> EP300/ETS/ETV/SP1 Expression Targets |
|  | NRG1 -> CREB/CREBBP/ELK/SRF/MYC Expression Targets |
|  | NRG1 -> TP53 Expression Target |
|  | NRG1 -> AP-1/ATF Expression Targets |
|  | NRG1 -> HIF1A Expression Target |
|  | NGF -> AP-1/TP53/MYC Expression Targets |
|  | NGF -> FOXO/MYCN/ELK-SRF Expression Targets |
|  | BDNF Expression Targets |
|  | NTF3 Expression Targets |
|  | NTF4 Expression Targets |
|  | NGF -> CREB/CEBPB/MEF2A Expression Targets |
|  | Adenosine Expression Targets |
|  | PDGFB -> AP-1/CREB/MYC Expression Target |
|  | PDGFC -> CREB Expression Target |
|  | PDGFD -> AP-1 Expression Targets |
|  | PDGF -> AP-1/CREB/CREBBP/MYC Expression Targets |
|  | KIT -> MITF Signaling |
|  | PDGFR -> AP-1/MYC Signaling |
|  | AVP/Gs -> CREB/ELK/SRF/AP-1/EGR Expression Targets |
|  | GNRH1 Expression Targets |
|  | NTS Expression Targets |
|  | GNRH2 Expression Targets |
|  | OXT Expression Targets |
|  | POMC Expression Targets |
|  | EDN1 Expression Targets |
|  | IFNA1/Gq Expression Targets |
|  | EDN3 Expression Targets |
|  | AVP/Gq -> CREB/ELK/SRF/AP-1/EGR Expression Targets |
|  | PRL/GHR -> NF/kB/ELK/SRF/MYC Expression Targets |
|  | PDCD1 -> AP-1 Expression Targets |
|  | PTPRC -> BCL6 Signaling |
|  | AngiopoietinR -> AP-1 Signaling |
|  | GDNF -> HSF1 Signaling |
|  | EphrinB -> MAPK/JUN/FOS Signaling |
|  | NTRK -> AP-1/CREB/ELK/SRF/MYC/SMAD3/TP53 Signaling |
|  | GAS6 -> AP-1/CREB Expression Targets |
|  | FIGF -> AP-1 Expression Target |
|  | FIGF -> NCOR2 Expression Target |
|  | KITLG -> AP-1/CREB/CREBBP/MYC Expression Targets |
|  | GDNF -> HSF1 Expression Targets |
|  | CSF1 -> AP-1/CREB/CREBBP/MYC Expression Targets |
|  | PGF -> AP-1/CREB/CREBBP/MYC Expression Targets |
|  | FLT3LG -> AP-1/CREB/CREBBP Expression Targets |
|  | Lysophosphatidic Acid/LPARs Signaling |
|  | Androgen Receptor non-Genomic Signaling |
|  | FOXO1 Signaling |
|  | MERTK Signaling |
|  | Ras Signaling |
|  | AMPK Signaling |
|  | AXL Receptor Tyrosine Kinase Signaling |
|  | mTOR Signaling |
|  | KRAS Signaling |
|  | VEGF Signaling |
|  | Estrogens/ESR1 non-Genomic Signaling |
|  | EGFR Signaling |
|  | ERK/MAPK Canonical Signaling |
|  | TCR -> AP-1 Expression Targets |
|  | T-Cell Receptor -> AP-1 Signaling |
|  | TGFA -> TP53 Expression Targets |
|  | TGFA -> HIF1A Expression Targets |
|  | TGFA/AP-1/ATF Expression Targets |
|  | TGFA -> CREB/CREBBP/ELK-SRF/MYC Expression Targets |
|  | LTA Expression Targets |
|  | TNF -> AP-1 Expression Targets |
|  | TNF -> ELK-SRF Expression Targets |
|  | TNF -> CREB Expression Targets |
|  | TNF -> TP53/ATF Expression Targets |
|  | TNFRSF1A -> AP-1/ATF/TP53 Signaling |
|  | TNFRSF1A -> CREB/ELK-SRF Signaling |
|  | Thrombopoietin -> AP-1/CREB/CREBBP/MYC Expression Targets |
|  | Thrombopoietin -> SP1 Expression Targets |
|  | IL1B -> NO Expression Targets |
|  | IL1B -> PGE2 Expression Targets |
|  | ROS in Angiotensin Mediated Cardiovascular Remodeling and Hypertrophy |
|  | PLAU -> ELK-SRF/AP-1 Expression Targets |
|  | VEGFA -> ATF/CREB/ELK-SRF Expression Targets |
|  | VEGFC -> ATF Expression Target |
|  | VEGFA -> AP-1/CREBBP/MYC Expression Targets |
|  | VEGFA -> NCOR2 Expression Target |
|  | VEGFR -> AP-1/CREB/MYC Signaling |
|  | VEGFR -> ATF/CREB/ELK-SRF Signaling |
|  | pathway 3 |
|  | ace2 |
|  | Model of PPARG signaling in psoriasis |
|  | Model of PPARG related pathways in psoriasis (short version) |
|  | 1\_Inflammation in keratinocytes in the pathology of acne |
|  | 2\_Hyperseborrhea in acne vulgaris |
|  | 2\_Acute phase initiation in atopic dermatitis |
|  | 2\_Interleukin-17 and interleukin-22 signaling in psoriasis |
|  | 1\_The onset of atopic dermatitis |
|  | 1\_TNF and IL1B provoke ECM degradation in osteoarthritis |
|  | 2\_Osteoblast-mediated bone demineralization |
|  | 2\_The role of endothelial cells in pyelonephritis |
|  | 1\_Local estrogen production stimulates ectopic endometrium proliferation |
|  | 3\_Enhanced angiogenesis in endometriosis |
|  | 1\_Disruption of luteinizing hormone and follicle-stimulating hormone secretion in PCOS |
|  | 2\_Pathogens stimulate mucins expression in the middle ear |
|  | 2\_Oxidized LDL causes endothelial dysfunction in atherosclerosis |
|  | 2\_Cardiomyocyte hypertrophy |
|  | 3\_Dysfunction of smooth muscle cells in hypertension |
|  | 2\_The intense proliferation and contractile activity of smooth muscle cells in pulmonary hypertension |
|  | 1\_Cigarette smoke causes dysfunction and production of inflammatory mediators in alveolar epithelial cells |
|  | 1\_Th2 cell response in asthma |
|  | 3\_1\_Mucus accumulation in asthma |
|  | 2\_1\_Eosinophilia and neutrophilia in asthma: eosinophil migration and activation |
|  | 2\_2\_Eosinophilia and neutrophilia in asthma: eosinophil apoptosis |
|  | 4\_2\_Airway smooth muscle cells proliferation and airway tissue remodeling |
|  | 4\_Mucin hyperproduction in goblet and mucous cells in COPD |
|  | 1\_CD4+ T cell and epithelial cell response in celiac disease |
|  | 2\_CD8+ T-cell response and mucosal damage in celiac disease |
|  | 1\_1\_Insulin synthesis and secretion: insulin processing and storage |
|  | 4\_1\_Insulin resistance: insulin signaling |
|  | 3\_Islet beta-cell destruction in T1D |
|  | 1\_Microangiopathy in diabetic neuropathy |
|  | 2\_1\_General pathways of thyroid hormone signaling |
|  | public list |
|  | New Pathway |
|  | KRAS->RAFs->MAPK1/3 |
|  | NRAS->RAFs->MAPK1/3 |
|  | Other RASs-RAFs-MAPK1/3 |
|  | mapk1 |
|  | New Pathway |
|  | mapk1 protein modificators |
|  | Interleukin-17 and interleukin-22 signaling in psoriasis |

---

|  |  |
| --- | --- |
| Group | Proteins Involved in Cowden Syndrome |
|  | Genes Associated with Syndromic Epilepsy |

---

|  |  |
| --- | --- |
| Source | GO component |
|  | ResNet |
|  | curated |

---

|  |  |
| --- | --- |
| MedScan ID | 5894 |

---

|  |  |
| --- | --- |
| LocusLink ID | 5894 |
|  | 110157 |
|  | 24703 |
|  | 19372 |
|  | 76876 |
|  | 30716 |
|  | 101411 |
|  | 106517 |
|  | 12906 |
|  | 104335 |
|  | 281441 |

---

|  |  |
| --- | --- |
| Alias | Raf I |
|  | sarcoma 3611 oncogene |
|  | 6430402F14 |
|  | v-raf-leukemia viral oncogene 1 |
|  | murine leukemia viral (v-raf-1) oncogene homolog 1 (3611-MSV) |
|  | cRAF-1 |
|  | C-Raf proto-oncogene, serine/threonine kinase |
|  | CMD1NN |
|  | RAS-GTP binding protein kinase |
|  | OTTMUSP00000022608 |
|  | AA990557 |
|  | KRAF\_HUMAN |
|  | Raf kinases |
|  | Raf kinase |
|  | kinase Raf 1 |
|  | murine sarcoma 3611 oncogene 1 |
|  | Oncogene RAF1 |
|  | RAF1 |
|  | c-Raf |
|  | protein kinase Raf |
|  | rapidly-accelerated fibrosarcoma-1 |
|  | Raf-1 proto-oncogene, serine/threonine kinase |
|  | OTTHUMP00000209389 |
|  | v-raf-1 murine leukemia viral oncogene homolog 1 |
|  | cRaf |
|  | murine sarcoma 3611 oncogene I |
|  | proto-oncogene c-RAF |
|  | v-raf-1 murine leukemia viral oncogene homolog I |
|  | MGC102375 |
|  | cRAF1 |
|  | Raf |
|  | proto oncogene proteins c raf |
|  | RAF-1 |
|  | BB129353 |
|  | Craf1 transforming gene |
|  | OTTHUMP00000207813 |
|  | 6430402F14Rik |
|  | c-Raf1 |
|  | raf oncogene |
|  | raf proto-oncogene serine/threonine protein kinase |
|  | OTTMUSP00000022609 |
|  | v-raf-1 leukemia viral oncogene 1 |
|  | protein kinase raf I |
|  | rapidly-accelerated fibrosarcoma (Raf)-1 |
|  | v-raf-1 murine leukemia viral oncogene-like protein 1 |
|  | Oncogene RAF I |
|  | RIKEN cDNA 6430402F14 gene |
|  | Raf 1 kinase |
|  | v-Raf |
|  | v-raf-1 leukemia viral oncogene I |
|  | NS5 |
|  | RAF proto-oncogene serine/threonine-protein kinase |
|  | D830050J10Rik |
|  | Craf1 transforming |
|  | v-raf-1 murine leukaemia viral oncogene homolog I |
|  | v-raf-1 murine leukaemia viral oncogene homolog 1 |
|  | murine leukaemia viral (v-raf-1) oncogene homolog 1 (3611-MSV) |
|  | v-raf-leukaemia viral oncogene 1 |
|  | v-raf-1 leukaemia viral oncogene I |
|  | v-raf-1 leukaemia viral oncogene 1 |
|  | protein kinase raf 1 |
|  | protein kinase raf-1 |
|  | c-Raf-1 |
|  | c-Raf-1 s |
|  | (RAF) |
|  | KRAF |
|  | (RAF1) |
|  | MIL |
|  | RAF1\_HUMAN |

---

|  |  |
| --- | --- |
| EC Number | 2.7.11.1 |
|  | 2.7.1.37 |
|  | 2.7.1.- |

---

|  |  |
| --- | --- |
| GO ID | 0005524 |
|  | 0004709 |
|  | 0010856 |
|  | 0008179 |
|  | 0019899 |
|  | 0046872 |
|  | 0031434 |
|  | 0046982 |
|  | 0004672 |
|  | 0004674 |
|  | 0031267 |
|  | 0000165 |
|  | 0000186 |
|  | 0007190 |
|  | 0006915 |
|  | 0030154 |
|  | 0071550 |
|  | 0060324 |
|  | 0007507 |
|  | 0035773 |
|  | 0045104 |
|  | 0034220 |
|  | 0043066 |
|  | 0008285 |
|  | 0043154 |
|  | 1902042 |
|  | 0031333 |
|  | 0048011 |
|  | 0030168 |
|  | 0033138 |
|  | 0045944 |
|  | 0006468 |
|  | 0035023 |
|  | 0042981 |
|  | 0045595 |
|  | 2000145 |
|  | 0001666 |
|  | 0035994 |
|  | 0007165 |
|  | 0035019 |
|  | 0002223 |
|  | 0048538 |
|  | 0030878 |
|  | 0042060 |
|  | 0005794 |
|  | 0005737 |
|  | 0005829 |
|  | 0005741 |
|  | 0005739 |
|  | 0016607 |
|  | 0005886 |
|  | 0031143 |
|  | 0017016 |
|  | 0016301 |
|  | 0000166 |
|  | 0016740 |
|  | 0001678 |
|  | 0035556 |
|  | 0016310 |
|  | 0001934 |
|  | 0016020 |
|  | 0005634 |
|  | 0008283 |
|  | 0005057 |
|  | 0004697 |
|  | 0038095 |
|  | 0007265 |
|  | 0007411 |
|  | 0007596 |
|  | 0007173 |
|  | 0008543 |
|  | 0045087 |
|  | 0008286 |
|  | 0007264 |
|  | 0007268 |
|  | 0055085 |
|  | 0048010 |
|  | 0016772 |
|  | 0007010 |
|  | 0007243 |
|  | 0005622 |
|  | 0008152 |
|  | 0005626 |
|  | 0023034 |
|  | 0008270 |
|  | 0019992 |
|  | 0005515 |
|  | 0007242 |
|  | 0051726 |
|  | 0009987 |
|  | 0004713 |
|  | 0006829 |
|  | 0000074 |
|  | 0050875 |

---

|  |  |
| --- | --- |
| KEGG ID | hsa:5894 |
|  | mmu:110157 |
|  | rno:24703 |

---

|  |  |
| --- | --- |
| MedLine Reference | 16396499 |
|  | 12237340 |
|  | 3550433 |
|  | 1889815 |
|  | 8318738 |
|  | 6320371 |
|  | 15075335 |
|  | 15674327 |
|  | 3616625 |
|  | 7590978 |
|  | 11751906 |
|  | 8816460 |
|  | 15890671 |
|  | 12223526 |
|  | 15302887 |
|  | 11597136 |
|  | 10704835 |
|  | 8702721 |
|  | 9779826 |
|  | 12477808 |
|  | 15618521 |
|  | 8707830 |
|  | 3003687 |
|  | 10440867 |
|  | 15666389 |
|  | 8939988 |
|  | 9790773 |
|  | 10433554 |
|  | 12466851 |
|  | 10205168 |
|  | 1376319 |
|  | 8649829 |
|  | 9115221 |
|  | 8566003 |
|  | 12360521 |
|  | 11427728 |
|  | 12691824 |
|  | 11217851 |
|  | 12133899 |
|  | 2193291 |
|  | 7987303 |
|  | 11076861 |
|  | 12626521 |
|  | 17097642 |
|  | 9207797 |
|  | 16301319 |
|  | 10585452 |
|  | 11733498 |
|  | 8753867 |
|  | 15041731 |
|  | 1611909 |
|  | 8603510 |
|  | 16803888 |
|  | 11933072 |
|  | 9153224 |
|  | 2341153 |
|  | 12391160 |
|  | 8035810 |
|  | 12954639 |
|  | 7628630 |
|  | 16332724 |
|  | 16861903 |
|  | 12127063 |
|  | 8661054 |
|  | 9931261 |
|  | 11604401 |
|  | 15736129 |
|  | 15662129 |
|  | 8641130 |
|  | 15845549 |
|  | 8692945 |
|  | 1572642 |
|  | 15753127 |
|  | 15886202 |
|  | 16565089 |
|  | 8876196 |
|  | 9823899 |
|  | 17081983 |
|  | 8125298 |
|  | 12244094 |
|  | 11296228 |
|  | 11296227 |
|  | 7517401 |
|  | 16964381 |
|  | 9234708 |
|  | 15836771 |
|  | 2475255 |
|  | 9281368 |
|  | 2758403 |
|  | 8349614 |
|  | 15314064 |
|  | 2993863 |
|  | 6690719 |
|  | 11971957 |
|  | 16465392 |
|  | 16737746 |
|  | 16883571 |
|  | 1992343 |
|  | 16086581 |
|  | 14623285 |
|  | 9819434 |
|  | 12925535 |
|  | 14654844 |
|  | 15856007 |
|  | 8900182 |
|  | 9710607 |
|  | 14636584 |
|  | 16527894 |
|  | 10775035 |
|  | 12149272 |
|  | 9693046 |
|  | 12717443 |
|  | 1690378 |
|  | 16266749 |
|  | 8929531 |
|  | 7906967 |
|  | 8929532 |
|  | 10922068 |
|  | 12865432 |
|  | 7766599 |
|  | 15289381 |
|  | 7629107 |
|  | 10531364 |
|  | 15657590 |
|  | 10837247 |
|  | 9099670 |
|  | 16909118 |
|  | 9446616 |
|  | 10064593 |
|  | 17397260 |
|  | 1765007 |
|  | 8349631 |
|  | 10523642 |
|  | 17554210 |
|  | 11042159 |
|  | 16364920 |
|  | 12429936 |
|  | 8157000 |
|  | 8910675 |
|  | 7774940 |
|  | 16707572 |
|  | 2539530 |
|  | 10498616 |
|  | 9624170 |
|  | 1639773 |
|  | 15211515 |
|  | 16575407 |
|  | 11306563 |
|  | 17428866 |
|  | 8048167 |
|  | 15782199 |
|  | 17277048 |
|  | 7782277 |
|  | 11447113 |
|  | 16109975 |
|  | 9315742 |
|  | 1738193 |
|  | 17535812 |
|  | 15356004 |
|  | 9867809 |
|  | 8084603 |
|  | 14688025 |
|  | 8036007 |
|  | 8393135 |
|  | 10497893 |
|  | 10869570 |
|  | 17064357 |
|  | 8575777 |
|  | 10373531 |
|  | 1846778 |
|  | 10207156 |
|  | 17603483 |
|  | 17603482 |
|  | 15664191 |
|  | 15703070 |
|  | 8095010 |
|  | 12801936 |
|  | 9001246 |
|  | 7939632 |
|  | 8835546 |
|  | 11756411 |
|  | 8408024 |
|  | 8104057 |
|  | 1302014 |
|  | 12620389 |
|  | 7644510 |
|  | 9852579 |
|  | 1322500 |
|  | 3003402 |
|  | 16413928 |
|  | 15741175 |
|  | 9689060 |
|  | 7896292 |
|  | 10329666 |
|  | 10783161 |
|  | 11908948 |
|  | 15591313 |
|  | 6308607 |
|  | 8455932 |
|  | 10523633 |
|  | 9373149 |
|  | 11226259 |
|  | 11788587 |
|  | 17243098 |
|  | 16432225 |
|  | 7649477 |
|  | 12538354 |
|  | 12663662 |
|  | 15067053 |
|  | 9553107 |
|  | 2463463 |
|  | 15485920 |
|  | 11784866 |
|  | 8325640 |
|  | 16569214 |
|  | 8812432 |
|  | 9523700 |
|  | 8889548 |
|  | 8321321 |
|  | 15899852 |
|  | 9671314 |
|  | 9038343 |
|  | 17462920 |
|  | 12010819 |
|  | 11997508 |
|  | 15489334 |
|  | 14706453 |
|  | 16679305 |
|  | 1783381 |
|  | 2878803 |
|  | 8385802 |
|  | 12901876 |
|  | 6324342 |
|  | 8290278 |
|  | 7935384 |
|  | 15102471 |
|  | 16365167 |
|  | 12087097 |
|  | 12975377 |
|  | 8063729 |
|  | 1645445 |
|  | 11325826 |
|  | 8756332 |
|  | 11853019 |
|  | 9767153 |
|  | 1612588 |
|  | 17332776 |
|  | 7908202 |
|  | 9488663 |
|  | 16862215 |
|  | 16239230 |
|  | 12123723 |
|  | 16141073 |
|  | 7896277 |
|  | 16141072 |
|  | 15710605 |
|  | 7905852 |
|  | 10454553 |
|  | 15711535 |
|  | 8530446 |
|  | 9696043 |
|  | 10969079 |
|  | 15451031 |
|  | 7731720 |
|  | 12490852 |
|  | 12532330 |
|  | 15653554 |
|  | 16537381 |
|  | 8710867 |
|  | 11409918 |
|  | 11157055 |
|  | 11005817 |
|  | 16029117 |
|  | 11134016 |
|  | 14576068 |
|  | 10917603 |
|  | 8394352 |
|  | 3399964 |
|  | 12374792 |
|  | 2955285 |
|  | 15849194 |
|  | 11171046 |
|  | 15670823 |
|  | 11350735 |
|  | 8999914 |
|  | 9230211 |
|  | 8499946 |
|  | 7730360 |
|  | 1572667 |
|  | 15305288 |
|  | 7594541 |
|  | 8777726 |
|  | 16709817 |
|  | 8530057 |
|  | 12851216 |
|  | 10576742 |
|  | 16172610 |
|  | 70317 |
|  | 16508002 |
|  | 12612072 |
|  | 15122343 |
|  | 16924233 |
|  | 12843393 |
|  | 15601844 |
|  | 15975997 |
|  | 10620507 |
|  | 11044439 |
|  | 15467832 |
|  | 12477932 |
|  | 15470083 |
|  | 10349636 |
|  | 8911690 |
|  | 8334704 |
|  | 12551923 |
|  | 8340392 |
|  | 9091312 |
|  | 7588749 |
|  | 15925322 |
|  | 8621729 |
|  | 1346779 |
|  | 8034307 |
|  | 16945999 |
|  | 14712229 |
|  | 7587388 |
|  | 3692486 |
|  | 12438425 |
|  | 12682854 |
|  | 10486249 |
|  | 7477354 |
|  | 1679528 |
|  | 11948406 |
|  | 7791872 |

---

|  |  |
| --- | --- |
| Organism | Homo sapiens {Organism urn:agi-taxid:9606} |
|  | Mus musculus {Organism urn:agi-taxid:10090} |
|  | Rattus norvegicus {Organism urn:agi-taxid:10116} |
|  | Homo sapiens |
|  | Mus musculus |
|  | Rattus norvegicus |
|  | Danio rerio |
|  | Bos taurus |

---

|  |  |
| --- | --- |
| Mouse chromosome position | 6 53.62 cM |
|  | 6 52.5 cM |

---

|  |  |
| --- | --- |
| OMIM ID | 164760 |
|  | 615916 |
|  | 611554 |
|  | 611553 |
|  | OMIM:164760 |

---

|  |  |
| --- | --- |
| Rat chromosome position | 4q42 |

---

|  |  |
| --- | --- |
| Hugo ID | 9829 |
|  | HGNC:9829 |

---

|  |  |
| --- | --- |
| Human chromosome position | 3p25.2 |
|  | 3p25 |

---

|  |  |
| --- | --- |
| Swiss-Prot Accession | L7RRS6 |
|  | P04049 |
|  | A0A0S2Z4L5 |
|  | P04049.1 |
|  | Q99N57 |
|  | Q99N57.2 |
|  | P11345 |
|  | P11345.1 |
|  | B0LPH8 |
|  | B2R5N3 |
|  | Q15278 |
|  | Q9UC20 |
|  | Q3UR68 |
|  | Q58E75 |
|  | Q91WH1 |
|  | Q99N58 |
|  | Q9QUU8 |
|  | B4E0X2 |
|  | R9PXU5 |
|  | Q8VI85 |
|  | Q99N73 |
|  | Q9CU36 |
|  | PASK\_HUMAN |
|  | RAF1\_RAT |
|  | RAF1\_HUMAN |
|  | RAF1\_MOUSE |

---

|  |  |
| --- | --- |
| PIR ID | A00637 |
|  | S60341 |
|  | A26126 |

---

|  |  |
| --- | --- |
| GenBank ID | NC\_000003 |
|  | NR\_148942 |
|  | NR\_148941 |
|  | NR\_148940 |
|  | XR\_001740227 |
|  | NM\_002880 |
|  | NP\_002871 |
|  | XM\_011533974 |
|  | XP\_011532276 |
|  | XM\_017006966 |
|  | XP\_016862455 |
|  | NM\_001354695 |
|  | NP\_001341624 |
|  | NM\_001354692 |
|  | NP\_001341621 |
|  | NM\_001354694 |
|  | NP\_001341623 |
|  | NM\_001354693 |
|  | NP\_001341622 |
|  | NM\_001354689 |
|  | NP\_001341618 |
|  | NM\_001354691 |
|  | NP\_001341620 |
|  | NM\_001354690 |
|  | NP\_001341619 |
|  | NG\_007467 |
|  | AC018500 |
|  | AC026170 |
|  | AH002959 |
|  | AAA60247 |
|  | AX696362 |
|  | CAD86886 |
|  | AY271661 |
|  | AAP03432 |
|  | CH471055 |
|  | EAW64134 |
|  | EAW64135 |
|  | EU332868 |
|  | ABY87557 |
|  | HI376400 |
|  | CBX55729 |
|  | JC006878 |
|  | CDL93489 |
|  | JX512459 |
|  | AGC09606 |
|  | KT585044 |
|  | M38134 |
|  | AK226028 |
|  | AK303561 |
|  | BAG64584 |
|  | AK303920 |
|  | BAG64848 |
|  | AK312248 |
|  | BAG35180 |
|  | BC018119 |
|  | AAH18119 |
|  | BQ221862 |
|  | BU683389 |
|  | BX648063 |
|  | DC342671 |
|  | KU178297 |
|  | ALQ33755 |
|  | KU178298 |
|  | ALQ33756 |
|  | KU178299 |
|  | ALQ33757 |
|  | X03484 |
|  | CAA27204 |
|  | P04049 |
|  | NC\_000072 |
|  | NM\_029780 |
|  | NP\_084056 |
|  | XM\_006505363 |
|  | XP\_006505426 |
|  | XM\_006505365 |
|  | XP\_006505428 |
|  | XM\_006505364 |
|  | XP\_006505427 |
|  | NM\_001356333 |
|  | NM\_001356334 |
|  | AB077821 |
|  | AC160032 |
|  | CH466523 |
|  | EDK99533 |
|  | EDK99534 |
|  | EDK99535 |
|  | JC006876 |
|  | CDL93488 |
|  | X55432 |
|  | AB052739 |
|  | BAB39527 |
|  | AB057655 |
|  | BAB39748 |
|  | AB057663 |
|  | BAB39743 |
|  | AB077145 |
|  | BAB83675 |
|  | AK018272 |
|  | BAB31142 |
|  | AK036317 |
|  | AK141745 |
|  | BAE24820 |
|  | AK148327 |
|  | AK189599 |
|  | AK190457 |
|  | AK191593 |
|  | AK193756 |
|  | AK193894 |
|  | AK199760 |
|  | AK205887 |
|  | AK215463 |
|  | BC015273 |
|  | AAH15273 |
|  | BC056439 |
|  | BC092040 |
|  | AAH92040 |
|  | BF455519 |
|  | Q99N57 |
|  | NC\_005103 |
|  | NM\_012639 |
|  | NP\_036771 |
|  | XM\_008763217 |
|  | XP\_008761439 |
|  | XM\_008763218 |
|  | XP\_008761440 |
|  | AC\_000072 |
|  | AABR07073061 |
|  | AAHX01032457 |
|  | AAHX01032458 |
|  | AC094445 |
|  | CH473964 |
|  | EDM02147 |
|  | EDM02148 |
|  | EDM02149 |
|  | BC062071 |
|  | AAH62071 |
|  | M15427 |
|  | AAA42001 |
|  | P11345 |
|  | XM\_005265359 |
|  | XP\_005265416 |
|  | XM\_005265358 |
|  | XP\_005265415 |
|  | XM\_011533975 |
|  | XP\_011532277 |
|  | XM\_005265357 |
|  | XP\_005265414 |
|  | XM\_005265355 |
|  | XP\_005265412 |
|  | XM\_005265360 |
|  | XP\_005265417 |
|  | NC\_018914 |
|  | AMYH02006294 |
|  | L00206 |
|  | L00207 |
|  | L00208 |
|  | L00209 |
|  | L00210 |
|  | L00211 |
|  | L00212 |
|  | M11376 |
|  | AC\_000028 |
|  | AAHY01056762 |
|  | AAHY01056763 |
|  | AAHY01056764 |
|  | AC\_000135 |
|  | ABBA01025197 |
|  | ABBA01025198 |
|  | ABBA01025199 |
|  | ABBA01025200 |
|  | XM\_005265356 |
|  | XP\_005265413 |
|  | AABR06032993 |
|  | AABR06032994 |
|  | AABR06032995 |
|  | XM\_003749820 |
|  | XP\_003749868 |
|  | NT\_022517 |
|  | NW\_001838877 |
|  | NW\_921651 |
|  | AC\_000046 |
|  | CR598160 |
|  | CR608506 |
|  | CR609564 |
|  | CR616511 |
|  | CR617502 |
|  | NT\_039353 |
|  | NW\_001030811 |
|  | Q8VI85 |
|  | Q99N73 |
|  | Q9CU36 |
|  | NW\_001084832 |
|  | NW\_047696 |
|  | Q58E75 |
|  | 33991673 |
|  | 81867258 |
|  | AB100685 |
|  | 148667118 |
|  | 148667119 |
|  | 148667117 |
|  | Q7ZTR8 |
|  | 50896407 |
|  | 206547 |
|  | 119584539 |
|  | 119584538 |
|  | 40363564 |
|  | 81903918 |
|  | M15428 |
|  | AA990557 |
|  | Q3UR68 |
|  | AAA42002 |
|  | 51464027 |
|  | 206546 |
|  | 206545 |
|  | 206544 |
|  | BG085782 |
|  | BB129353 |
|  | BC054662 |
|  | 26085159 |
|  | CAA57035 |
|  | 496091 |
|  | 18497289 |
|  | Q76I87 |
|  | NW\_000264 |
|  | AB095292 |
|  | 18497290 |
|  | 6981458 |
|  | 34190937 |
|  | 30409368 |
|  | 81882448 |
|  | 13429987 |
|  | 74706531 |
|  | Q90458 |
|  | 51464120 |
|  | 125653 |
|  | NT\_094510 |
|  | BAD11990 |
|  | 13429988 |
|  | 51717211 |
|  | NP\_571514 |
|  | AB116254 |
|  | 125651 |
|  | 13383316 |
|  | 13383317 |
|  | NW\_001512500 |
|  | NT\_086636 |
|  | NC\_007134 |
|  | BG073005 |
|  | 74202437 |
|  | 34858854 |
|  | 82241273 |
|  | 149049693 |
|  | 149049695 |
|  | 35841 |
|  | 149049694 |
|  | 35842 |
|  | AK142706 |
|  | 41224402 |
|  | 534977 |
|  | Q99N58 |
|  | 4506401 |
|  | 18150195 |
|  | 18150196 |
|  | 44885322 |
|  | 82239922 |
|  | 38303884 |
|  | Q15278 |
|  | 38303885 |
|  | 30016945 |
|  | 30016944 |
|  | Q6BE22 |
|  | 27884315 |
|  | BAC76320 |
|  | Q7ZZW3 |
|  | 18157433 |
|  | 18157434 |
|  | 12857903 |
|  | 12857902 |
|  | AB104854 |
|  | NM\_131439 |
|  | X81128 |
|  | 81903252 |
|  | BAD08233 |
|  | 62024525 |
|  | 34098686 |
|  | 15929695 |
|  | 17390263 |
|  | 52486392 |
|  | 15929694 |
|  | Q76FQ3 |
|  | 82235645 |
|  | BAC55895 |
|  | 82216957 |
|  | 190837 |
|  | 40254798 |
|  | BAD06254 |
|  | BAD34647 |
|  | 82132302 |
|  | 66762 |
|  | 82093361 |
|  | AB098701 |
|  | AB098700 |
|  | BC045925 |
|  | 81867927 |
|  | 123788363 |

---

|  |  |
| --- | --- |
| Swiss-Prot ID | RAF1\_HUMAN |
|  | RAF1\_MOUSE |
|  | RAF1\_RAT |

---

|  |  |
| --- | --- |
| Cell Localization | Cytoplasm |
|  | Cell membrane |
|  | Mitochondrion |
|  | Nucleus |
|  | Mitochondria |

---

|  |  |
| --- | --- |
| Ensembl ID | ENSG00000132155 |
|  | ENSP00000401888.2 |
|  | ENST00000442415.6 |
|  | ENSP00000251849.4 |
|  | ENST00000251849.8 |
|  | ENSP00000398591.2 |
|  | ENST00000432427.2 |
|  | ENSMUSG00000000441 |
|  | ENSMUSP00000000451.7 |
|  | ENSMUST00000000451.13 |
|  | ENSMUSP00000108571.1 |
|  | ENSMUST00000112949.7 |
|  | ENSRNOG00000010153 |
|  | ENSRNOP00000013831.5 |
|  | ENSRNOT00000013831.6 |
|  | ENSP00000251849 |
|  | ENST00000251849 |
|  | ENSP00000398591 |
|  | ENST00000432427 |
|  | ENSMUSP00000000451 |
|  | ENSMUST00000000451 |
|  | ENSMUSP00000108571 |
|  | ENSMUST00000112949 |
|  | ENSRNOP00000013831 |
|  | ENSRNOT00000013831 |

---

|  |  |
| --- | --- |
| MGI ID | MGI:97847 |
|  | 97847 |
|  | 1924126 |
|  | 2141729 |
|  | 2146790 |
|  | 1277195 |

---

|  |  |
| --- | --- |
| RGD ID | 3531 |

---

|  |  |
| --- | --- |
| Unigene ID | Hs.159130 |
|  | Mm.184163 |
|  | Rn.33262 |
|  | Dr.75082 |
|  | Hs.257266 |
|  | Dr.122213 |
|  | Mm.347772 |

---

|  |  |
| --- | --- |
| Homologene ID | 48145 |

---

|  |  |
| --- | --- |
| Shape | Sickle-vertex |

---

|  |  |
| --- | --- |
| IPI ID | IPI00021786 |
|  | IPI00900312 |
|  | IPI00193150 |
|  | IPI00118101 |
|  | IPI00830899 |
|  | IPI00788857 |
|  | IPI00789533 |
|  | IPI00792701 |
|  | IPI00793356 |

---

|  |  |
| --- | --- |
| Microarray ID | 94264\_at |

---

|  |  |
| --- | --- |
| Homo sapiens Chromosome position | 3p25 |

---

|  |  |
| --- | --- |
| Ensembl Transcript ID | ENST00000251849 |

---

|  |  |
| --- | --- |
| ENSEMBL | ENSG00000132155 |

---

|  |  |
| --- | --- |
| FunctionalClass | protein kinase |
|  | protein heterodimerization |
|  | zinc ion binding |
|  | mitogen-activated protein kinase kinase binding |
|  | metal ion binding |
|  | nucleotide binding |
|  | MAP kinase kinase kinase |
|  | receptor signaling protein |
|  | diacylglycerol binding |
|  | kinase |
|  | protein binding |
|  | protein-tyrosine kinase |
|  | protein serine/threonine kinase |
|  | transferase |
|  | ATP binding |

---

|  |  |
| --- | --- |
| Hugo Symbol | RAF1 |

---

|  |  |
| --- | --- |
| Bos taurus Chromosome position | 22 |

---

|  |  |
| --- | --- |
| Mus musculus Chromosome position | 6 52.5 cM |
|  | 6 |

---

|  |  |
| --- | --- |
| KEGG pathway | Renal cell carcinoma |
|  | Gap junction |
|  | Chronic myeloid leukemia |
|  | Non-small cell lung cancer |
|  | Pancreatic cancer |
|  | Colorectal cancer |
|  | MAPK signaling pathway |
|  | VEGF signaling pathway |
|  | Melanoma |
|  | Insulin signaling pathway |
|  | Prostate cancer |
|  | Melanogenesis |
|  | Natural killer cell mediated cytotoxicity |
|  | Focal adhesion |
|  | Long-term potentiation |
|  | Regulation of actin cytoskeleton |
|  | Dorso-ventral axis formation |
|  | ErbB signaling pathway |
|  | Glioma |
|  | Endometrial cancer |
|  | GnRH signaling pathway |
|  | Long-term depression |
|  | Fc epsilon RI signaling pathway |

---

|  |  |
| --- | --- |
| ProteinAtlas SubcellularLocation | Main location: Nucleus but not nucleoli; Other location: ; Expression type: Staining; Reliability: Non-supportive |

---

|  |  |
| --- | --- |
| Rattus norvegicus Chromosome position | 4q42 |

---

|  |  |
| --- | --- |
| Ensembl Gene ID | ENSG00000132155 |

---

|  |  |
| --- | --- |
| ProteinAtlas NormalTissue | Tissue: hippocampus; Cell type: neuronal cells; Level: Moderate; Expression type: Staining; Reliability: Uncertain |
|  | Tissue: cerebellum; Cell type: Purkinje cells; Level: Moderate; Expression type: Staining; Reliability: Uncertain |
|  | Tissue: cerebral cortex; Cell type: neuronal cells; Level: Weak; Expression type: Staining; Reliability: Uncertain |
|  | Tissue: thyroid gland; Cell type: glandular cells; Level: Negative; Expression type: Staining; Reliability: Uncertain |
|  | Tissue: vulva/anal skin; Cell type: epidermal cells; Level: Weak; Expression type: Staining; Reliability: Uncertain |
|  | Tissue: salivary gland; Cell type: glandular cells; Level: Negative; Expression type: Staining; Reliability: Uncertain |
|  | Tissue: hippocampus; Cell type: glial cells; Level: Negative; Expression type: Staining; Reliability: Uncertain |
|  | Tissue: liver; Cell type: bile duct cells; Level: Weak; Expression type: Staining; Reliability: Uncertain |
|  | Tissue: cerebral cortex; Cell type: glial cells; Level: Weak; Expression type: Staining; Reliability: Uncertain |
|  | Tissue: testis; Cell type: Leydig cells; Level: Negative; Expression type: Staining; Reliability: Uncertain |
|  | Tissue: lymph node; Cell type: non-germinal center cells; Level: Weak; Expression type: Staining; Reliability: Uncertain |
|  | Tissue: rectum; Cell type: glandular cells; Level: Negative; Expression type: Staining; Reliability: Uncertain |
|  | Tissue: liver; Cell type: hepatocytes; Level: Low; Expression type: APE; Reliability: Low |
|  | Tissue: lung; Cell type: macrophages; Level: Low; Expression type: APE; Reliability: Low |
|  | Tissue: lung; Cell type: pneumocytes; Level: Low; Expression type: APE; Reliability: Low |
|  | Tissue: adrenal gland; Cell type: glandular cells; Level: Low; Expression type: APE; Reliability: Low |
|  | Tissue: appendix; Cell type: glandular cells; Level: Low; Expression type: APE; Reliability: Low |
|  | Tissue: appendix; Cell type: lymphoid tissue; Level: Low; Expression type: APE; Reliability: Low |
|  | Tissue: bone marrow; Cell type: hematopoietic cells; Level: Low; Expression type: APE; Reliability: Low |
|  | Tissue: breast; Cell type: glandular cells; Level: Low; Expression type: APE; Reliability: Low |
|  | Tissue: bronchus; Cell type: respiratory epithelial cells; Level: Low; Expression type: APE; Reliability: Low |
|  | Tissue: cerebellum; Cell type: cells in granular layer; Level: Medium; Expression type: APE; Reliability: Low |
|  | Tissue: cerebellum; Cell type: cells in molecular layer; Level: Low; Expression type: APE; Reliability: Low |
|  | Tissue: cerebellum; Cell type: Purkinje cells; Level: Medium; Expression type: APE; Reliability: Low |
|  | Tissue: cerebral cortex; Cell type: glial cells; Level: Low; Expression type: APE; Reliability: Low |
|  | Tissue: cerebral cortex; Cell type: neuronal cells; Level: Low; Expression type: APE; Reliability: Low |
|  | Tissue: cervix, uterine; Cell type: glandular cells; Level: Low; Expression type: APE; Reliability: Low |
|  | Tissue: cervix, uterine; Cell type: squamous epithelial cells; Level: Low; Expression type: APE; Reliability: Low |
|  | Tissue: colon; Cell type: glandular cells; Level: Low; Expression type: APE; Reliability: Low |
|  | Tissue: duodenum; Cell type: glandular cells; Level: Low; Expression type: APE; Reliability: Low |
|  | Tissue: epididymis; Cell type: glandular cells; Level: Low; Expression type: APE; Reliability: Low |
|  | Tissue: esophagus; Cell type: squamous epithelial cells; Level: Low; Expression type: APE; Reliability: Low |
|  | Tissue: fallopian tube; Cell type: glandular cells; Level: Low; Expression type: APE; Reliability: Low |
|  | Tissue: gall bladder; Cell type: glandular cells; Level: Medium; Expression type: APE; Reliability: Low |
|  | Tissue: heart muscle; Cell type: myocytes; Level: Low; Expression type: APE; Reliability: Low |
|  | Tissue: hippocampus; Cell type: glial cells; Level: Low; Expression type: APE; Reliability: Low |
|  | Tissue: hippocampus; Cell type: neuronal cells; Level: Medium; Expression type: APE; Reliability: Low |
|  | Tissue: kidney; Cell type: cells in glomeruli; Level: Low; Expression type: APE; Reliability: Low |
|  | Tissue: kidney; Cell type: cells in tubules; Level: Low; Expression type: APE; Reliability: Low |
|  | Tissue: lateral ventricle; Cell type: glial cells; Level: Medium; Expression type: APE; Reliability: Low |
|  | Tissue: lateral ventricle; Cell type: neuronal cells; Level: Medium; Expression type: APE; Reliability: Low |
|  | Tissue: liver; Cell type: bile duct cells; Level: Low; Expression type: APE; Reliability: Low |
|  | Tissue: lymph node; Cell type: germinal center cells; Level: Low; Expression type: APE; Reliability: Low |
|  | Tissue: lymph node; Cell type: non-germinal center cells; Level: Low; Expression type: APE; Reliability: Low |
|  | Tissue: nasopharynx; Cell type: respiratory epithelial cells; Level: Medium; Expression type: APE; Reliability: Low |
|  | Tissue: oral mucosa; Cell type: squamous epithelial cells; Level: Low; Expression type: APE; Reliability: Low |
|  | Tissue: ovary; Cell type: follicle cells; Level: Low; Expression type: APE; Reliability: Low |
|  | Tissue: ovary; Cell type: ovarian stroma cells; Level: Low; Expression type: APE; Reliability: Low |
|  | Tissue: pancreas; Cell type: exocrine glandular cells; Level: Low; Expression type: APE; Reliability: Low |
|  | Tissue: pancreas; Cell type: islets of Langerhans; Level: Low; Expression type: APE; Reliability: Low |
|  | Tissue: parathyroid gland; Cell type: glandular cells; Level: Low; Expression type: APE; Reliability: Low |
|  | Tissue: placenta; Cell type: decidual cells; Level: Medium; Expression type: APE; Reliability: Low |
|  | Tissue: placenta; Cell type: trophoblastic cells; Level: Low; Expression type: APE; Reliability: Low |
|  | Tissue: prostate; Cell type: glandular cells; Level: Low; Expression type: APE; Reliability: Low |
|  | Tissue: rectum; Cell type: glandular cells; Level: Low; Expression type: APE; Reliability: Low |
|  | Tissue: salivary gland; Cell type: glandular cells; Level: Low; Expression type: APE; Reliability: Low |
|  | Tissue: seminal vesicle; Cell type: glandular cells; Level: Low; Expression type: APE; Reliability: Low |
|  | Tissue: skeletal muscle; Cell type: myocytes; Level: Low; Expression type: APE; Reliability: Low |
|  | Tissue: skin; Cell type: epidermal cells; Level: Low; Expression type: APE; Reliability: Low |
|  | Tissue: small intestine; Cell type: glandular cells; Level: Low; Expression type: APE; Reliability: Low |
|  | Tissue: smooth muscle; Cell type: smooth muscle cells; Level: Low; Expression type: APE; Reliability: Low |
|  | Tissue: spleen; Cell type: cells in red pulp; Level: Low; Expression type: APE; Reliability: Low |
|  | Tissue: spleen; Cell type: cells in white pulp; Level: Low; Expression type: APE; Reliability: Low |
|  | Tissue: stomach, lower; Cell type: glandular cells; Level: Low; Expression type: APE; Reliability: Low |
|  | Tissue: stomach, upper; Cell type: glandular cells; Level: Low; Expression type: APE; Reliability: Low |
|  | Tissue: testis; Cell type: cells in seminiferus ducts; Level: Low; Expression type: APE; Reliability: Low |
|  | Tissue: testis; Cell type: Leydig cells; Level: Low; Expression type: APE; Reliability: Low |
|  | Tissue: thyroid gland; Cell type: glandular cells; Level: Low; Expression type: APE; Reliability: Low |
|  | Tissue: tonsil; Cell type: germinal center cells; Level: Low; Expression type: APE; Reliability: Low |
|  | Tissue: tonsil; Cell type: non-germinal center cells; Level: Low; Expression type: APE; Reliability: Low |
|  | Tissue: tonsil; Cell type: squamous epithelial cells; Level: Low; Expression type: APE; Reliability: Low |
|  | Tissue: urinary bladder; Cell type: urothelial cells; Level: Medium; Expression type: APE; Reliability: Low |
|  | Tissue: uterus, post-menopause; Cell type: cells in endometrial stroma; Level: Low; Expression type: APE; Reliability: Low |
|  | Tissue: uterus, post-menopause; Cell type: glandular cells; Level: Low; Expression type: APE; Reliability: Low |
|  | Tissue: uterus, pre-menopause; Cell type: cells in endometrial stroma; Level: Low; Expression type: APE; Reliability: Low |
|  | Tissue: uterus, pre-menopause; Cell type: glandular cells; Level: Low; Expression type: APE; Reliability: Low |
|  | Tissue: vagina; Cell type: squamous epithelial cells; Level: Low; Expression type: APE; Reliability: Low |
|  | Tissue: vulva/anal skin; Cell type: epidermal cells; Level: Low; Expression type: APE; Reliability: Low |

---

|  |  |
| --- | --- |
| LocatorString | pmquery:egfr+transactivation |

---
